# The association between dietary micronutrient patterns and odds of diabetic nephropathy: A case–control study

**DOI:** 10.1002/fsn3.3306

**Published:** 2023-03-31

**Authors:** Niki Bahrampour, Atieh Mirzababaei, Faezeh Abaj, Dorsa Hosseininasab, Cain C. T. Clark, Khadijeh Mirzaei

**Affiliations:** ^1^ Department of Nutrition, Science and Research Branch Islamic Azad University (SRBIAU) Tehran Iran; ^2^ Department of Community Nutrition, School of Nutritional Sciences and Dietetics Tehran University of Medical Sciences (TUMS) Tehran Iran; ^3^ Centre for Intelligent Healthcare Coventry University Coventry CV1 5FB UK

**Keywords:** chronic kidney disease, diabetes, diabetic nephropathy, fat‐soluble vitamins, micronutrient patterns, minerals

## Abstract

Uncontrolled diabetes can lead to diabetic nephropathy (DN). The aim of the study was to investigate the relationship between different dietary micronutrient patterns and risk of DN in women. This was a case–control study. One hundred and five patients had DN (defined as urinary mg of albumin per gram of creatinine ≥30 mg/g) were chosen as the case and 105 women without DN were chosen as control. Dietary intakes were assessed by a semi‐quantitative food frequency questionnaire. Principal component analysis with varimax rotation was used to derive the micronutrient patterns. Patterns were divided into two groups of lower and higher than median. Logistic regression was used to discern and find the odds ratio (ORs) of DN, and its 95% confidence interval (CI) based on the micronutrient patterns in crude and adjusted model. Three patterns which were included, (1) mineral patterns such as chromium, manganese, biotin, vitamin B6, phosphorus, magnesium, selenium, copper, zinc, potassium, and iron, (2) water‐soluble vitamin patterns such as vitamin B5, B2, folate, B1, B3, B12, sodium and C, and (3) fat‐soluble vitamin patterns such as calcium, vitamin K, beta carotene, alpha tocopherol, alpha carotene, vitamin E, and vitamin A, were extracted. An inverse relationship was found between risk of DN and following mineral patterns and fat‐soluble vitamin patterns in adjusted model (ORs = 0.51 [95% CI 0.28–0.95], *p* = .03) and (ORs = 0.53 [95% CI 0.29–0.98], *p* = .04), respectively. No relationship was seen between water‐soluble vitamin patterns and risk of DN in crude and adjusted model but the significance was decreased in adjusted model. The risk of DN was 47% decreased after high adherence of fat‐soluble vitamin patterns. In addition, we saw a 49% decrease of risk of DN in high adherence group of mineral patterns. The findings confirm that renal‐protective dietary patterns can reduce risk of DN.

## Graphical abstract

The risk of diabetic nephropathy was 47% decreased after high adherence of fat‐soluble vitamin patterns. In addition, we saw a 49% decrease of risk of DN in high adherence group of mineral patterns. The findings emphasize that dietary sources of renal‐protective nutrients should be encouraged among the general population.

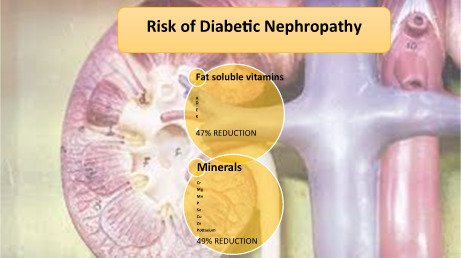

## 1 INTRODUCTION

Chronic kidney disease (CKD) affects almost 10% of people in the world (Piccoli et al., 2018) and 40% of diabetic patients are diagnosed with CKD (Aziz et al., 2021). Although the prevalence of CKD is higher in men than women, due to the effect of sex hormones on the development of CKD, it is necessary to next investigate about this disease among women (Fanelli et al., 2017). Uncontrolled diabetes can lead to diabetic nephropathy (DN), where its progression risks are hypertension, high blood glucose, and lipid profiles. Most patients with type 2 diabetes are prone to macroalbuminuria (urine level ≥300 mg/24 h) or nephropathy. Early recognition of DN can help to reduce complications and mortality (Lee et al., 2021).

Dietary intake can play an indispensable effect in the progression of DN, both independently and dependently. For example, low sodium diets (50–70 mmol daily) and fluid consumption restrictions are effective in controlling DN (Borrelli et al., 2020). In addition, lower consumption of potassium, protein (0.6–0.8 g/kg/day), and phosphorus can be helpful in treating DN (Kim, 2014); while adequate intake of zinc is also recommended (Galindo et al., 2020). Most diabetic patients typically experience higher low‐density lipoprotein (LDL) and total cholesterol (TC; Lim, 2014), where high‐fat diets can induce renal damage through macrophage activation (Kim et al., 2019). In addition, injection of vitamin D is significantly associated with decreasing pain in patients with DN (Basit et al., 2016). Serum uric acid, which is the product of purines is usually high in DN patients, and lower consumption of purine‐rich foods, such as peas, lentils, beans, spinach, mushrooms, and cauliflower, and higher consumption of vitamin C can elicit positive outcomes (Beydoun et al., 2018). Furthermore, higher fructose accumulation, which is high in fruits, could contribute to the development of DN through metabolism of uric acid and glucose (Bjornstad et al., 2015). Interestingly, a recent study found that supplementation with vitamins B6, B9, and B12 exacerbated the DN (Thornalley & Rabbani, 2010), while magnesium deficiency was shown to be strongly related to DN and insulin resistance (Ranjith Kumar & Santhosh, 2015).

Dietary micronutrient patterns are the combination of vitamins and minerals from the consumption of foods. These patterns can elucidate the relationships and interactions of such nutrients on people's health. Therefore, the aim of the study was to investigate the relationship between dietary micronutrient patterns and risk of DN in women.

## 2 MATERIALS AND METHODS

### 2.1 Study design and study setting

This was a case–control study designed at the Kowsar Diabetes Clinic in Semnan, Iran. The one‐to‐one matching method was used to recruit 105 patients with DN cases. Nephropathy was defined as urinary mg of albumin per gram of creatinine (ACR) ≥30 mg/g, via a random spot urine sample (Aziz et al., 2021). The selection was among women, who had type 2 diabetes, were if they were aged between 30 and 65 years, had fasting blood glucose (FBG) ≥126 mg/dL, or 2‐h post‐load blood glucose ≥200 mg/dL; glycosylated hemoglobin (HbA1c) ≥6.5% and these values last for 3–10 years without any past diseases, such as cancer, liver dysfunction, and autoimmune disorders (Molitch et al., 2004). Control subjects were not different in most clinical characteristics from those observed in the case subjects. They were not with DN.

### 2.2 Data collection

A demographic questionnaire was used to discern the general variables, including age, diabetes duration, medical history, and medicine history. Furthermore, both systolic and diastolic blood pressure were assessed, after the subjects rested for 15 min.

### 2.3 Measurement anthropometric

Weight (kg) was measured while the participants had light clothing and were unshod, using a Seca scale, to the nearest 0.1 kg. Height (cm) was measured, with participants standing erect beside a wall‐mounted stadiometer (SECA 217), to the nearest 0.1 cm. In addition, WC (cm) was measured at the midpoint of the lowest rib and the hip bone, while BMI (kg/m^2^) was calculated by dividing weight by the square of height.

### 2.4 Physical activity assessment

Physical activity of participants was evaluated via the short form of the international physical activity questionnaire (IPAQ). The IPAQ has previously been validated in this country (Craig et al., 2003).

### 2.5 Biochemical markers

A random spot urine sample was collected by enzyme‐linked immunosorbent assay [sensitivity 0.001 mg/L; coefficient of variation (CV) 4.5e7.6%] to examine urinary albumin excretion. Blood levels of hemoglobin (Hb), fasting glucose (FBG), blood glucose (BS), HbA1, triglycerides (TG), low‐ and high‐density lipoprotein (LDL, HDL), serum vitamin D, TC, creatinine (Cr), and blood urea nitrogen (BUN) were collected via the past 3‐month medical history.

### 2.6 Dietary intake assessment and dietary micronutrient patterns

Dietary intakes were assessed by a 147‐item semi‐quantitative food frequency questionnaire (FFQ), considering the past year, via face‐to‐face interview. The reliability and validity of FFQ have previously been approved in this country (Esfahani et al., 2010; Willett et al., 1997). All foods and beverages were converted to grams and analyzed using Nutritionist‐IV software. Participants with total energy intakes lower than 500 kcal or more than 3500 kcal were excluded from the study. Dietary vitamins and minerals were converted into three patterns.

### 2.7 Statistical analysis

**FIGURE 1:**
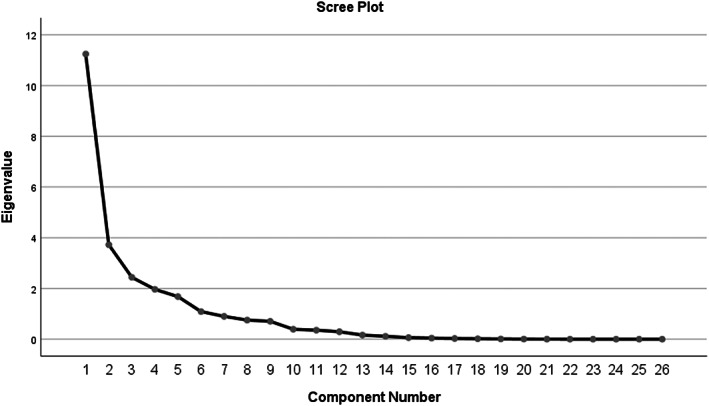
Scree plot of the micronutrients and the extracted principal components.

The normality of variables was evaluated using Kolmogorov–Smirnov test. Principal component analysis (PCA) with varimax rotation was used to derive the micronutrient patterns. Factor scores of subjects for each of the extracted factors were calculated by summing the frequency of consumption, multiplied by factor loadings across nutrients. Based on the scree plot (eigenvalue >1), three patterns were extracted (Figure 1). For each pattern, the median was calculated by the sum of the nutrients’ intakes and divided into two groups of lower and higher than median. Then, the patterns were adjusted for energy intake. The chi‐square test and independent samples *T*‐test were used to find the differences in general and dietary intakes of subjects among lower and higher adherence of median of each micronutrient pattern. Finally, logistic regression was used to discern the odds ratio (ORs) of DN, and its 95% confidence interval (CI), based on the micronutrient patterns in crude and adjusted models. The adjusted model was adjusted for energy intake, age, physical activity, hemoglobin, serum vitamin D3, and albumin. *p* < .05 was, a priori, considered to represent statistical significance. Data analyses were conducted with SPSS (version 20.0; SPSS Inc).

## 3 RESULTS

### 3.1 Baseline characteristics of the participants

General variables of the participants are presented across case and control groups and micronutrient patterns quantiles in Tables 1 and 2, respectively. Serum albumin, HbA1c, LDL, creatinine, medication use of angiotensin receptor blockers (ARBs), and angiotensin‐converting enzyme inhibitors (ACEIs) were significantly different between the case and control group (*p* < .05).Overall, the total energy intake of cases and controls were 1407.64 ± 254.51 and 1452.26 ± 320.95 kcal, respectively. The height and percent of HbA1c were significantly different between lower and higher adherence of fat‐soluble vitamin patterns (*p* < .05). Incidence of DN decreased among quantiles of mineral and fat‐soluble vitamin patterns (*p* < .05).

**TABLE 1:** Characteristics of participants across case and control groups.

Variables	Control (*n* = 105)	Case (*n* = 105)	*p* value*
Age (year)	55.41 ± 7.14	55.33 ± 7.04	.94
Diabetes duration (year)	7.56 ± 2.17	7.60 ± 2.21	.88
SBP (mmHg)	129.04 ± 98.88	126.59 ± 17.27	.80
DBP (mmHg)	80.10 ± 11.76	82.80 ± 13.09	.12
Anthropometric measurements			
Body weight (kg)	71.589 ± 11.50	73.400 ± 13.83	.30
Height (cm)	161.17 ± 5.91	160.68 ± 6.29	.56
BMI (kg/m^2^)	27.510 ± 4.39	28.686 ± 4.74	.06
Biochemical measurements			
Albumin (mg/dL)	8.37 ± 6.76	14.40 ± 11.94	**<.001**
Hb (mg/dL)	12.630 ± 1.22	12.610 ± 1.37	.91
FBS (mg/dL)	154.19 ± 45.03	167.10 ± 50.62	**.05**
BS (mg/dL)	207.10 ± 54.35	217.75 ± 53.23	.15
HbA1c (%)	8.03 ± 1.29	8.66 ± 1.41	**<.001**
Cholesterol (mg/dL)	175.38 ± 32.42	185.15 ± 38.12	**.05**
TG (mg/dL)	162.25 ± 57.91	167.26 ± 65.68	.56
LDL (mg/dL)	94.60 ± 29.47	106.86 ± 31.77	**<.001**
HDL (mg/dL)	46.37 ± 9.25	45.05 ± 9.26	.30
Cr (mg/dL)	0.87 ± 0.17	0.92 ± 0.16	**.03**
BUN (mg/dL)	15.17 ± 3.86	15.79 ± 4.55	.29
PA (met‐h/w)			
Low (>600)	37 (17.6)	31 (14.8)	.12
Moderate (600–3000)	28 (13.4)	42 (20)	
High (<3000)	40 (19)	32 (15.2)	
Medical history			
CVD history	23 (11)	24 (11.4)	.86
Medication history			
ARBs	45 (21.4)	60 (28.6)	**.03**
ACEIs	21 (10)	44 (21)	**.001**
Beta blockers	18 (8.6)	20 (9.5)	.56
Metformin	104 (49.5)	104 (49.5)	.75
Sulfonylurea	62 (29.5)	71 (33.8)	.19
Insulin	35 (16.7)	26 (12.4)	.17

*Note*: Independent sample *T*‐test and chi‐square test were used.

Continuous variables were shown as mean ± SD and qualitative variables were shown *n* (%).

*p* < .05 was considered significant and showed by bold font.

Abbreviation: ACEIs, angiotensin‐converting enzyme inhibitors; ARBs, angiotensin receptor blockers; BMI, body mass index; BS, blood sugar; BUN, blood urea nitrogen; CR, creatinine; CVD, cardiovascular disease; DBP, diastolic blood pressure; FBS, fasting blood sugar; Hb, hemoglobin; HDL, high‐density lipoprotein; LDL, low‐density lipoprotein; met, metabolic equivalent; PA, physical activity; SBP, systolic blood pressure; TG, triglycerides.

* Adjusted for energy intake.

**TABLE 2:** General and biochemical characteristics of participants across micronutrient patterns.

Variables	Mineral pattern^a^			Water‐soluble vitamin pattern			Fat‐soluble vitamin pattern		
	Low	High	*p* value	Low	High	*p* value	Low	High	*p* value
Age (y)	55.66 ± 7.08	55.09 ± 7.09	.56	55.85 ± 6.80	54.85 ± 7.36	.31	54.70 ± 7.53	55.95 ± 6.64	.20
Diabetes duration (y)	7.41 ± 2.16	7.75 ± 2.20	.27	7.72 ± 2.19	7.43 ± 2.17	.33	7.65 ± 2.13	7.52 ± 2.23	.66
SBP (mmHg)	130.71 ± 98.94	124.91 ± 16.49	.55	123.45 ± 16.75	132.61 ± 101.17	.35	124.45 ± 15.99	130.70 ± 95.51	.53
DBP (mmHg)	81.84 ± 12.13	81.06 ± 12.87	.65	81.00 ± 12.39	81.94 ± 12.63	.59	81.02 ± 12.25	81.81 ± 12.72	.65
Anthropometric measurements									
Body weight (kg)	71.49 ± 11.67	73.50 ± 13.67	.25	72.01 ± 13.11	73.03 ± 12.31	.56	72.64 ± 11.70	72.37 ± 13.58	.88
Height (cm)	160.94 ± 6.08	160.90 ± 6.13	.96	160.81 ± 6.04	161.05 ± 6.18	.78	162.02 ± 5.88	159.98 ± 6.14	**.02**
BMI (kg/m^2^)	27.56 ± 4.58	28.64 ± 4.57	.09	28.06 ± 4.53	28.14 ± 4.68	.91	27.72 ± 4.70	28.42 ± 4.50	.27
Biochemical measurements									
Albumin (g/dL)	10.27 ± 7.74	12.50 ± 12.01	.11	10.91 ± 8.55	11.91 ± 11.66	.48	12.44 ± 11.28	10.49 ± 9.01	.17
Vitamin D3 (mm/dL)	26.61 ± 16.05	28.61 ± 19.77	.42	29.75 ± 18.26	25.26 ± 17.48	.07	27.10 ± 17.02	28.05 ± 18.85	.70
Hb (mg/dL)	12.54 ± 1.31	12.70 ± 1.28	.39	12.75 ± 1.28	12.48 ± 1.30	.14	12.50 ± 1.24	12.72 ± 1.33	.21
FBS (mg/dL)	158.41 ± 48.15	162.89 ± 48.43	.50	162.02 ± 51.60	159.14 ± 44.43	.67	165.54 ± 43.90	156.45 ± 51.48	.17
BS (mg/dL)	210.39 ± 54.58	214.46 ± 53.46	.59	211.62 ± 52.93	213.31 ± 55.26	.82	218.57 ± 47.79	207.15 ± 58.39	.13
HbA1c (%)	8.25 ± 1.40	8.45 ± 1.37	.29	8.27 ± 1.37	8.43 ± 1.41	.41	8.57 ± 1.43	8.15 ± 1.32	**.03**
Cholesterol (mg/dL)	178.55 ± 36.20	181.98 ± 35.15	.49	178.71 ± 34.95	181.98 ± 36.47	.51	183.89 ± 33.34	177.16 ± 37.36	.17
TG (mg/dL)	162.82 ± 56.74	166.69 ± 66.73	.65	159.36 ± 53.97	170.68 ± 69.24	.19	161.64 ± 60.12	167.42 ± 63.39	.50
LDL (mg/dL)	100.68 ± 30.77	100.78 ± 31.73	.98	97.40 ± 31.81	104.39 ± 30.21	.10	103.74 ± 29.83	98.14 ± 32.20	.20
HDL (mg/dL)	46.42 ± 9.38	45.00 ± 9.12	.27	45.59 ± 9.19	45.84 ± 9.37	.85	45.55 ± 9.39	45.85 ± 9.18	.81
Cr (mg/dL)	0.90 ± 0.18	0.90 ± 0.16	.91	0.91 ± 0.17	0.88 ± 0.16	.22	0.89 ± 0.16	0.90 ± 0.17	.76
BUN (mg/dL)	15.10 ± 3.94	15.87 ± 4.47	.18	15.97 ± 4.32	14.95 ± 4.06	.08	16.00 ± 4.73	15.04 ± 3.69	.10
Nephropathy *n* (%)	45 (42.9)	60 (57.1)	**.03**	59 (56.2)	46 (43.8)	.26	56 (53.3)	49 (46.7)	**.03**
PA (MET/Min/week)									
Low	30 (44.1)	38 (55.9)	.39	37 (54.4)	31 (45.6)	.87	29 (42.6)	39 (57.4)	.68
Moderate	39 (55.7)	31 (44.3)		35 (50)	35 (50)		32 (45.7)	38 (54.3)	
High	36 (50)	36 (50)		38 (52.8)	34 (47.2)		36 (50)	36 (50)	
CVD history *n* (%)	23 (48.9)	24 (51.1)	.86	25 (53.2)	22 (46.8)	.90	25 (53.2)	22 (46.8)	.27
Medication history									
ARBs	51 (48.6)	54 (51.4)	.67	57 (54.3)	48 (45.7)	.58	51 (48.6)	54 (51.4)	.48
ACEIs	34 (52.3)	31 (47.7)	.65	34 (52.3)	31 (47.7)	.98	36 (55.4)	29 (44.6)	.07
Beta blockers	22 (57.9)	16 (42.1)	.35	19 (50)	19 (50)	.60	20 (52.6)	18 (47.4)	.36
Metformin	105 (50.5)	103 (49.5)	.49	108 (51.9)	100 (48.1)	.49	97 (46.6)	111 (53.4)	.50
Sulfonylurea	69 (51.9)	64 (48.1)	.47	69 (51.9)	64 (48.1)	.84	67 (50.4)	66 (49.6)	.11
Insulin	32 (52.5)	29 (47.5)	.64	33 (54.1)	28 (45.9)	.75	26 (42.6)	35 (57.4)	.50

*Note*: Independent sample *T*‐test and chi‐square test were used.

Continuous variables were shown as mean ± SD and qualitative variables were shown *n* (%).

*p* < .05 was considered significant and showed by bold font.

Abbreviations: ACEIs, angiotensin‐converting enzyme inhibitors; ARBs, angiotensin receptor blockers; BMI, body mass index; BS, blood sugar; BUN, blood urea nitrogen; CR, creatinine; CVD, cardiovascular disease; DBP, diastolic blood pressure; FBS, fasting blood sugar; Hb, hemoglobin; HDL, high‐density lipoprotein; LDL, low‐density lipoprotein; met, metabolic equivalent; PA, physical activity; SBP, systolic blood pressure; TG, triglycerides.

^a^ Patterns were adjusted for energy intake.

### 3.2 Dietary intakes of the subjects across micronutrient groups

We summarized total dietary intakes, vitamins, and minerals across quantiles of each micronutrient pattern in Table 3. In mineral and fat‐soluble vitamin patterns, the energy intake was reduced between quantiles of patterns (*p* < .05). Dietary intakes of protein, fat, cholesterol, saturated fatty acids (SFA), monounsaturated fatty acids (MUFA), oleic acid, beta carotene, alpha carotene, vitamin E, alpha tocopherol, vitamin K, C, B2, B12, biotin, and calcium were statistically lower after adjusting for energy intake (*p* < .05). However, magnesium, zinc, manganese, and chromium intake were increasing in this pattern. Consumption of MUFA, oleic acid, vitamin A, alpha carotene, vitamin E, alpha tocopherol, B6, biotin, phosphorus, magnesium, zinc, manganese, chromium, and potassium was significantly different in the water‐soluble vitamin pattern (*p* < .05). Fiber, linolenic acid, B1, B2, B9, B12, calcium, and sodium intakes were increasing across quantiles in this pattern. Finally, in fat‐soluble vitamin patterns, significant decreases were seen in protein, cholesterol, SFA, B1, B2, B3, B12, B5, biotin, phosphorus, magnesium, zinc, manganese, chromium, calcium, sodium, copper, iron, and selenium intakes (*p* < .05) across quantiles; while consumption of linolenic acid, beta carotene, alpha carotene, vitamin E, alpha tocopherol, vitamin K, and vitamin A were increased in the mentioned pattern. Furthermore, extracting patterns are shown in Table 4. Pattern 1 included minerals such as chromium, manganese, biotin, vitamin B6, phosphorus, magnesium, selenium, copper, zinc, potassium, and iron. Pattern 2 included water‐soluble vitamins such as vitamins B5, B2, folate, B1, B3, B12, sodium, and C. The last pattern consisted of fat‐soluble vitamins such as calcium, vitamin K, beta carotene, alpha tocopherol, alpha carotene, vitamin E, and vitamin A.

**TABLE 3:** dietary intakes of participants across micronutrient patterns.

Variable	Mineral pattern^a^			Water‐soluble vitamin pattern			Fat‐soluble vitamin pattern		
	Low	High	*p*‐value	Low	High	*p*‐value	Low	High	*p*‐value
Energy intake (kcal)	1479.07 ± 381.16	1289.30 ± 199.22	**.002**	1449.64 ± 292.78	1408.30 ± 286.42	.30	1473.66 ± 266.13	1392.43 ± 304.88	**.04**
Protein (g)	45.58 ± 11.05	43.37 ± 6.83	.21	46.47 ± 7.41	47.56 ± 10.87	.39	48.85 ± 9.53	45.39 ± 8.66	**.006**
Carbohydrate (g)	258.48 ± 72.62	221.70 ± 40.73	**.002**	257.35 ± 58.19	244.64 ± 53.32	.10	257.98 ± 48.49	245.56 ± 61.62	.11
Fat (g)	34.62 ± 12.03	31.21 ± 4.37	**.05**	33.54 ± 9.53	32.35 ± 5.85	.27	33.72 ± 7.58	32.33 ± 8.32	.21
Fiber (g)	38.42 ± 9.04	36.92 ± 4.49	.27	36.46 ± 7.21	40.58 ± 9.54	**<.001**	37.47 ± 8.13	39.24 ± 8.99	.13
Cholesterol (g)	10.61 ± 12.46	6.02 ± 4.68	**.01**	5.77 ± 7.77	7.81 ± 8.57	.07	7.96 ± 8.53	5.69 ± 7.80	**.04**
SFA (g)	6.44 ± 2.37	5.84 ± 1.05	**.08**	6.33 ± 1.83	6.08 ± 1.42	.26	6.50 ± 1.53	5.96 ± 1.71	**.01**
MUFA (g)	11.61 ± 5.19	10.20 ± 1.68	**.05**	11.48 ± 3.63	10.58 ± 2.36	**.03**	11.18 ± 2.69	10.95 ± 3.45	.60
PUFA (g)	10.68 ± 2.33	10.56 ± 1.48	.74	10.35 ± 2.79	10.77 ± 1.80	.20	10.62 ± 2.94	10.49 ± 1.76	.69
Oleic acid (g)	11.25 ± 5.00	9.86 ± 1.55	**.05**	11.13 ± 3.49	10.26 ± 2.23	**.03**	10.83 ± 2.59	10.62 ± 3.29	.61
Linoleic acid (g)	9.46 ± 2.07	9.37 ± 1.34	.79	9.33 ± 2.59	9.57 ± 1.63	.43	9.59 ± 2.76	9.33 ± 1.53	.39
Linolenic acid (g)	1.02 ± 0.28	0.99 ± 0.21	.57	0.85 ± 0.33	0.99 ± 0.21	**<.001**	0.86 ± 0.34	0.97 ± 0.23	**.009**
Fat‐soluble vitamins									
A (RAE)	23.08 ± 15.24	23.22 ± 9.87	.95	24.20 ± 14.20	20.62 ± 12.07	**.05**	15.95 ± 12.02	28.11 ± 11.76	**<.001**
Beta carotene (mg)	17.97 ± 13.10	14.34 ± 3.30	**.04**	17.02 ± 8.88	15.44 ± 5.67	.13	13.14 ± 3.64	18.96 ± 8.90	**<.001**
Alpha carotene (mg)	2.54 ± 6.34	0.40 ± 0.77	**.01**	1.31 ± 4.36	0.39 ± 0.63	**.03**	0.35 ± 0.41	1.32 ± 4.33	**.02**
E (μg)	4.07 ± 2.15	3.34 ± 0.45	**.01**	4.52 ± 1.79	3.58 ± 0.78	**<.001**	3.83 ± 0.93	4.28 ± 1.80	**.02**
Alpha tocopherol (mg)	1.98 ± 1.02	1.52 ± 0.29	**.001**	1.95 ± 0.75	1.63 ± 0.38	**<.001**	1.69 ± 0.45	1.89 ± 0.73	**.02**
K (μg)	14.28 ± 8.68	11.93 ± 1.60	**.04**	13.55 ± 5.91	12.58 ± 3.71	.16	11.76 ± 2.42	14.23 ± 6.23	**<.001**
Water‐soluble vitamins									
C (mg)	12.56 ± 6.67	7.46 ± 3.07	**<.001**	10.14 ± 5.45	10.69 ± 6.12	.48	9.64 ± 5.51	11.06 ± 5.93	.07
B1 (mg)	1.65 ± 0.40	1.55 ± 0.30	.14	1.64 ± 0.23	1.74 ± 0.41	**.03**	1.79 ± 0.37	1.60 ± 0.27	**<.001**
B2 (mg)	0.98 ± 0.25	0.90 ± 0.15	**.04**	0.93 ± 0.14	1.01 ± 0.23	**.003**	1.04 ± 0.20	0.91 ± 0.16	**<.001**
B3 (mg)	15.65 ± 3.57	14.56 ± 2.57	.07	15.88 ± 2.28	16.26 ± 3.65	.36	17.11 ± 3.35	15.16 ± 2.35	**<.001**
B6 (mg)	0.66 ± 0.12	0.69 ± 0.07	.10	0.81 ± 0.11	0.72 ± 0.15	**<.001**	0.76 ± 0.16	0.77 ± 0.12	.60
B9 (μg)	405.96 ± 121.85	368.18 ± 91.74	.07	355.71 ± 57.70	415.13 ± 117.42	**<.001**	392.61 ± 110.42	376.62 ± 80.67	.16
B12 (μg)	0.22 ± 0.23	0.14 ± 0.08	**.01**	0.13 ± 0.13	0.17 ± 0.16	**.03**	0.17 ± 0.16	0.13 ± 0.13	**.02**
B5 (mg)	2.22 ± 0.56	2.13 ± 0.41	.33	2.46 ± 0.55	2.46 ± 0.77	.96	2.63 ± 0.76	2.31 ± 0.53	**<.001**
Biotin (mg)	12.50 ± 2.45	15.28 ± 2.06	**<.001**	18.80 ± 5.12	16.20 ± 5.57	**.001**	18.82 ± 7.12	16.48 ± 3.16	**.002**
Minerals									
Phosphorus (mg)	799.04 ± 151.99	814.61 ± 91.88	.52	938.54 ± 135.74	871.98 ± 185.38	**.003**	934.24 ± 163.34	883.33 ± 162.18	**.02**
Magnesium (mg)	291.66 ± 48.31	327.35 ± 33.26	**<.001**	372.24 ± 73.97	337.45 ± 72.46	**.001**	366.45 ± 87.75	346.43 ± 61.20	**.05**
Zinc (mg)	6.99 ± 1.34	7.53 ± 0.83	**.01**	8.44 ± 2.67	7.92 ± 1.67	**.09**	8.66 ± 2.93	7.78 ± 1.34	**.005**
Copper (mg)	1.43 ± 0.21	1.46 ± 0.14	.39	1.53 ± 0.22	1.56 ± 0.29	.31	1.60 ± 0.28	1.50 ± 0.22	**.01**
Manganese (mg)	6.22 ± 1.01	7.53 ± 1.03	**<.001**	8.66 ± 1.57	7.62 ± 1.77	**<.001**	8.50 ± 1.69	7.88 ± 1.75	**.01**
Selenium (μg)	106.01 ± 24.69	110.17 ± 31.34	.45	124.67 ± 20.72	119.05 ± 33.69	.14	131.86 ± 32.17	113.52 ± 19.79	**<.001**
Chromium (mg)	0.14 ± 0.04	0.18 ± 0.03	**<.001**	0.25 ± 0.07	0.19 ± 0.06	**<.001**	0.23 ± 0.09	0.21 ± 0.06	**.01**
Calcium (mg)	411.95 ± 87.94	382.60 ± 45.91	**.03**	391.39 ± 66.54	417.58 ± 77.36	**.009**	418.38 ± 76.77	391.39 ± 67.29	**.007**
Iron (mg)	14.09 ± 2.46	13.69 ± 1.96	.35	14.99 ± 2.00	14.80 ± 2.78	.57	15.37 ± 2.47	14.50 ± 2.27	**.008**
Sodium (mg)	3311.54 ± 836.09	3306.65 ± 876.48	.97	3313.58 ± 901.21	3757.74 ± 1054.44	**.001**	3766.88 ± 987.02	3317.53 ± 967.66	**.001**
Potassium (mg)	1540.89 ± 393.55	1577.37 ± 210.47	.54	1778.59 ± 403.16	1629.92 ± 361.92	**.006**	1674.90 ± 343.91	1736.03 ± 425.66	.25

*Note*: Data are presented as mean ± standard deviation and resulted from independent sample *T*‐test.

*p* < .05 was considered significant and showed by bold font.

Abbreviation: MUFA, monounsaturated fatty acids; PUFA, polyunsaturated fatty acids; RAE, retinol activity equivalents; SFA, saturated fatty acids.

^a^ Patterns were adjusted for energy intake.

**TABLE 4:** Principal factor loading of micronutrient intakes.

Nutrients	Mineral pattern	Water‐soluble vitamin pattern	Fat‐soluble vitamin pattern
Chromium (mg)	0.916		
Manganese (mg)	0.865		
Biotin (mg)	0.836		
Vitamin B6 (mg)	0.824		
Phosphorus (mg)	0.824		
Magnesium (mg)	0.794		
Selenium (μg)	0.668		
Copper (mg)	0.650		
Vitamin B5 (mg)		0.470	
Zinc (mg)	0.587		
Potassium (mg)	0.540		
Vitamin B2 (mg)		0.875	
Folate (μg)		0.854	
Vitamin B1 (mg)		0.846	
Vitamin B3 (mg)		0.795	
Calcium (mg)			0.324
Iron (mg)	0.651		
Vitamin B12 (μg)		0.529	
Sodium (mg)		0.361	
Vitamin C (mg)		0.334	
Vitamin K (μg)			0.866
Beta carotene (mg)			0.856
Alpha tocopherol (mg)			0.827
Alpha carotene (mg)			0.823
Vitamin E (mg)			0.634
Vitamin A (RAE)			0.425
Percent of variance explained	28.99	22.65	15.30

Abbreviation: RAE, retinol activity equivalents.

Extraction method: PCA. Rotation Method: Varimax with Kaiser Normalization. a. Rotation converged in six iterations.

### 3.3 The association between micronutrient patterns and risk of DN

An inverse relationship was found between the risk of DN and mineral and fat‐soluble vitamin patterns in crude model (ORs for DN were 0.56 [95% CI 0.32–0.97]) and (ORs for DN were 0.56 [95% CI 0.32–0.97]), *p* = .03, respectively (Table 5). This negative association was remained in both mentioned patterns after adjusting for confounding variables (ORs 0.51 [95% CI 0.28–0.95]) and (ORs 0.53 [95% CI 0.29–0.98]), *p* < .05. In other words, the risk of DN was 47% decreased with high adherence of fat‐soluble vitamin pattern. In addition, we saw a 49% reduction of risk of DN with high adherence of the mineral patterns. No relationship was seen between the water‐soluble vitamin patterns and risk of DN in both crude and adjusted models.

**TABLE 5:** ORs and 95% CIs of diabetic nephropathy based on micronutrient patterns among cases and controls.

Variables	Reference (low)	Mineral pattern		Water‐soluble vitamin pattern		Fat‐soluble vitamin pattern	
		High	*p*‐value	High	*p*‐value	High	*p*‐value
Crude	1.00 (ref)	0.56 (0.32–0.97)	**.03**	0.73 (0.42–1.26)	.26	0.56 (0.32–0.97)	**.03**
Model 1	1.00 (ref)	0.51 (0.28–0.95)	**.03**	0.60 (0.33–1.10)	.09	0.53 (0.29–0.98)	**.04**

*Note*: Logistic regression was used. Low and high are presented as lower and higher adherence of median.

ORs (95% CI) is shown across patterns. *p* value < .05 was showed by bold font.

Model 1: Adjusted for energy intake, age, physical activity, hemoglobin, serum vitamin D3, and albumin.

## 4 DISCUSSION

In the present study, we investigated the association between dietary micronutrient patterns and the prevalence of DN. Accordingly, the main finding of the current study is that a diet rich in fat‐soluble vitamins and minerals is negatively associated with the presence of DN.

In the present study, participants who had higher adherence to fat‐soluble vitamin and mineral patterns had a lower risk of DN. In line with our study, Mazidi et al, indicated that diets rich in vitamins and trace elements are negatively associated with the risk of CKD (Mazidi et al., 2018). Another study, published in 2015, reported an inverse association between vitamin intake and CKD occurrence (Cheung et al., 2015). Moreover, an Australian cross‐sectional study showed that the intake of magnesium and folate decreased the risk of CKD by 40%–45% (Strippoli et al., 2011).

In our study, no association was observed between water‐soluble vitamin patterns and the risk of DN. However, in contrast to our study, Farhadnejad et al, reported that individuals with a higher intake of vitamin C had a reduced risk of CKD (Farhadnejad et al., 2016); additionally suggesting that individuals with a high intake of sodium had an increased risk of CKD (Farhadnejad et al., 2016).

DN is a prevalent microvascular complication of diabetes with an occurrence of nearly 30%–40% (Anders et al., 2018). DN is a leading cause of ESRD, renal transplantation, and mortality in patients with diabetes mellitus (DM; Anders et al., 2018); however, the complete pathogenic mechanism of DN is still unknown (Hu et al., 2019). Micronutrients are posited to play a protective role in the risk of CKD by reducing inflammatory markers containing interleukin 6, total homocysteine, and CRP (de Oliveira Otto et al., 2011). Indeed, the concentrations of some vitamins in blood of patients with DM have been shown to be lower than normal (Ahn et al., 2011; Thornalley et al., 2007). The lower concentrations of several vitamins in blood have been attributed to enhanced renal clearances of these vitamins, possibly because of the impaired reabsorption processes among patients with DM (Iwakawa et al., 2016). Thornalley et al, indicated that the renal clearance of vitamin B1 was higher in patients with T2DM, and concentrations of vitamin B1 in plasma negatively correlated with the renal clearance of vitamin B1 (Thornalley et al., 2007). Larkin et al, expressed that glucose‐induced reduced expression of thiamine transporters in the tubular epithelium may mediate renal mishandling of thiamine in DM (Larkin et al., 2012). Nevertheless, Fukui et al, did not report any significant differences in the renal clearance of vitamin B1 between patients with T2DM and normal controls (Iwakawa et al., 2016). Moreover, Shibata noted that renal clearances of vitamin E and some water‐soluble vitamins were higher in diabetic rats than in controls (Shibata et al., 1997).

Insulin resistance is a major issue in most T2DM patients, and vitamin D deficiency has been shown to result in insulin resistance and metabolic syndrome, especially concomitant to renal diseases and cardiovascular complications (Garbossa & Folli, 2017). Vitamin E is another fat‐soluble vitamin, which is well known for its antioxidant capacity; moreover, it also functions on the cell cycle, cell signaling, lipid metabolism, and inflammation (Gray et al., 2011). The underlying mechanistic action might include numerous pathways; for instance, its antioxidant capacity, where vitamin E alters IRS1 phosphorylation, and thus, impacts insulin signaling (Gray et al., 2011). Additionally, vitamin E was found to directly regulate gene expression such as PPAR‐δ, which plays a crucial role in insulin sensitivity (Landrier et al., 2009).

Several mineral substances are activating cofactors and coenzymes for metabolism control, oxidative stress, and genetic transcription. The deficiency of mineral substances has been shown to have a significant association with T2DM (Guo et al., 2020). An adequate selenium intake can act as an insulin‐mimetic to attenuate diabetes, with the role of reducing glucose and insulin tolerance, and therefore, preventing hepatic insulin resistance (Zhou et al., 2013). Indeed, chromium plays a vital role in glucose metabolism by augmenting the binding of insulin to INSR (Cefalu & Hu, 2004), where mechanisms underlying this beneficial function of chromium might partly be explained by the enhancement of GLUT2 expression and the activation of the PI3K/AKT pathway in skeletal muscle (Panchal et al., 2017). Zinc is a significant component of enzymes that play important roles in regulating insulin sensitivity and glucose homeostasis (Russell et al., 2016). Recent research indicated that, in patients with T2DM, the concentrations of zinc in plasma and tissues are generally lower (Russell et al., 2016). Another substance, magnesium, was suggested to reduce the risk of cardiovascular diseases in T2DM patients. However, not all the minerals are good for patients with DN; for instance, high sodium intake leads to a higher risk of hypertension and cardiovascular diseases (Zhao et al., 2016). Moreover, sodium intake enhances natriuresis via the PPAR‐δ/SGLT2 pathway and subsequently regulates glucose metabolism of T2DM patients (Zhao et al., 2016). Restricted sodium intake can lead to improvements in blood pressure in such patients. Although, high salt intake and urinary protein excretion have been shown to be related to annual creatinine clearance decline in patients with DN (Kanauchi et al., 2015). Throughout a weakening in renal function, potassium excretion is decreased leading to an accumulation in body tissues (Sulaiman, 2019). Hence, potassium intake, particularly from foods such as grains, potatoes, corn, soybean, nuts, tomatoes, banana, kiwi, etc., should be restricted (Sulaiman, 2019). Phosphorus excretion is also decreased throughout chronic kidney impairment leading to increased blood phosphorus levels (Sulaiman, 2019).

To the best of our knowledge, this is the first study to have assessed the association between micronutrient patterns and the risk of DN in a case–control design. However, our study has some limitations. The case–control nature of the study precludes cause‐and‐effect conclusions. In addition, there might be small errors in the dietary assessment, mostly due to (mis)recalling the data and misclassification errors by using FFQ. Moreover, our study only included women, and thus, results are not generalizable to men.

## 5 CONCLUSION

In conclusion, the results of our investigation suggest that higher intakes of several micronutrients, such as vitamins E, D, magnesium, and selenium decrease the risk of DN, whereas high intake of sodium is associated with an increased risk of incident DN. Our findings emphasize that dietary sources of renal‐protective nutrients should be encouraged among the general population. However, whether this knowledge can be exploited for DN prevention purposes must be ascertained in follow‐up studies.

## FUNDING INFORMATION

Tehran University of Medical Sciences and Health Services funded and supported the present study (Grant number: 94‐04‐161‐31155).

## CONFLICT OF INTEREST STATEMENT

The authors declare that there is no competing interest.

## ETHICAL APPROVAL

This research was conducted according to the Declaration of Helsinki. The study protocol was approved by the ethics committee of Tehran University of Medical Sciences (Ethic Number: IR.TUMS.REC.1395.2644), and by the ethics committee of Semnan University of Medical Sciences (Ethic Number: IR.SEMUMS.REC.1395.66).

## INFORMED CONSENT

Written informed consent was obtained from all subjects and/or their legal guardian(s).

## CONSENT FOR PUBLICATION

All authors listed approved the final manuscript and consent for publication.

## Data Availability

The data are not publicly available because of containing information that could compromise the privacy of the research. The datasets used and analyzed during the current study will be available from the corresponding author, Khadijeh Mirzaei, upon reasonable request.
